# In Situ Super-Resolution Imaging of Telomeres with DNA-PAINT

**DOI:** 10.1021/acsomega.2c05752

**Published:** 2022-10-31

**Authors:** Yuanyuan Liu, Xiangyu Ye, Zhuyuan Wang, Shenfei Zong, Yiping Cui

**Affiliations:** Advanced Photonics Center, Southeast University, Nanjing, Jiangsu210096, China

## Abstract

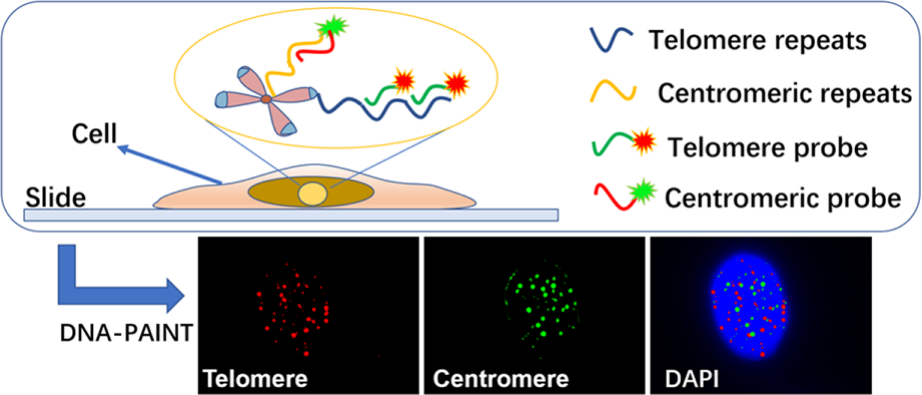

Telomeres are located at the ends of chromosomes and play an important role in maintaining the integrity of chromosomes and controlling the cycle of cell division. Studies have shown that abnormal telomere length may lead to the occurrence of some diseases. Therefore, accurate measurement of telomere length will be helpful for the prediction and diagnosis of related diseases. DNA point accumulation for imaging in nanoscale topography (PAINT) is an optical super-resolution technology that relies on the instantaneous binding of the fluorescent DNA imaging strand to the target epitope. Here, we present the first demonstration of DNA-PAINT-based in situ super-resolution imaging of telomeres as well as centromeres. For DNA-PAINT imaging, Cy5-labeled telomere DNA (5′-Cy5-TTTTTCCCTAACCCTAA-3′) and Cy3-labeled centromere DNA (5′-Cy3-TTTTTAGCTTCTGTCTAGTTT-3′) are utilized as the imager strands. Through an improved permeabilization strategy that we proposed, the imager strands can bind with intracellular telomeres and centromeres with high specificity, realizing super-resolution imaging of telomeres and centromeres. To check the applicability of DNA-PAINT in evaluating telomere length, we conducted an experiment using azidothymidine (AZT)-treated tumor cells as the imaging target. The DNA-PAINT imaging results clearly revealed the telomerase inhibition effect of AZT. Compared with single-molecule localization microscopy (SMLM) with peptide nucleic acid (PNA)-based fluorescence in situ hybridization (FISH), our method has the advantages of low cost, low toxicity, and simple equipment. Such a DNA-PAINT-based imaging strategy holds great potential in measuring telomere length with high accuracy, which would play an important role in the study of telomere-related diseases such as cancer.

## 1 Introduction

It is widely accepted that cancer cells require unlimited replicative potential in order to generate macroscopic tumors. Telomeres protecting the ends of chromosomes are centrally involved in the capability for unlimited proliferation.^1,2^ The telomeres composed of repetitive TTAGGG sequences in mammals, which shorten progressively in nonimmortalized cells, propagate in culture and eventually lose the ability to protect the ends of chromosomal DNAs from end-to-end fusions.^3^ There is clear evidence that telomeres are involved in cellular senescence and diseases of premature aging in humans.^4^ In addition, there is a link between the general health of humans and telomere length. Telomere shortening leads to the decline of biological tissue function, which increases the probability of disease.^5,6^ Telomerase, the specialized DNA polymerase that adds telomere repeat segments to the ends of telomeric DNA, is almost absent in nonimmortalized cells but expressed at functionally significant levels in the vast majority of spontaneously immortalized cells, including human cancer cells.^7^ Therefore, the detection of telomere length and telomerase can be helpful for cancer diagnosis.^2,8^

The rapid development of optical microscopy in the past 300 years has promoted human understanding in the biological field. However, the existence of the optical diffraction limit makes the traditional optical microscope only reach the spatial resolution of 200 nm at most.^9^ During the past two decades, several technologies have been invented to realize optical super-resolution imaging up to molecular-scale resolution inside cells.^10^ A variety of imaging modalities achieve image resolution beyond the diffraction limit by controlling the state of fluorophores such that only a small subset of them are detectable at any given time, including nonlinear structured illumination microscopy (SIM), stimulated emission depletion (STED) microscopy, photo-activated localization microscopy (PALM), and stochastic optical reconstruction microscopy (STORM).^11−15^ Among them, PALM and STORM are both based on single-molecule localization microscopy (SMLM).^16^ The SMLM technique requires that the fluorescence signals keep switching between bright and dark states.^17^ When sufficiently low-intensity activating light is applied to the sample, only a randomly sparse subset of fluorophores are activated to the on state at any time, allowing these molecules to be individually imaged and localized. In this method, many frames of wide-field images are collected, the positions of fluorophores are localized, and a super-resolved image is reconstructed from the frame sequences.^18^ In contrast to illumination-based modes, SMLM technology has no special instrumentation requirements. However, we need to carefully adjust the type of dye, labeling density, and buffer conditions to achieve the appropriate blinking behavior of the dye. As a result, SMLM must choose probes with controllable fluorescence properties, which is a drawback of this super-resolution imaging method.^19^

DNA point accumulation for imaging in nanoscale topography (DNA-PAINT) is a new super-resolution imaging method developed on the base of SMLM.^11^ DNA-PAINT relies on transient hybridization of a fluorophore-conjugated DNA imager strand with a target-associated reverse complementary DNA docking strand.^20^ Typically, the docking strand is immobilized on the biological target of interest, and the imager strand is labeled with an organic dye and diffuses freely in the imaging buffer. Then, since their sequences are complementary, the imager strand can bind to the docking strand. During their combined state, the camera is able to detect enough photons and output a strong signal. After the two strands are bound for a period of time, they dissociate, resulting in a dark state. Therefore, DNA-PAINT relies on the hybridization and dissociation of the two DNA strands to achieve photoswitching. What’s more, the timing of binding can be adjusted by changing the imaging buffer, the length of the strand, and the influx rate of the imager strand.^21^ Therefore, DNA-PAINT does not have strict requirements on the nature of the fluorophore, allowing us to choose brighter fluorophores to achieve ultrahigh resolution.^22^ More importantly, DNA-PAINT can be applied to achieve accurate counting, which is called qPAINT (quantitative PAINT).^23^ By analyzing the predictable binding kinetics between the imager and docking strands, qPAINT is able to count the number of targets. The qPAINT method is virtually unaffected by photobleaching and enables imaging over extended periods of time, resulting in higher precision. DNA-PAINT has been used to image telomere repeats produced by telomere elongation reaction and further quantify telomerase extracted from tumor cells by qPAINT method.^24^ To speed up imaging, FRET-PAINT microscopy has been developed and used to study the structure of telomeres.^25^

Herein, we present a method for in situ high-resolution imaging of telomeres within the nucleus using DNA-PAINT. In our experiments, the imager strand is a Cy5-labeled DNA strand complementary to telomeres in the nucleus. The docking strand is the telomeric DNA strand in the nucleus. As an inner control, DNA-PAINT imaging of centromeres was also conducted using Cy3-labeled imager strands. First, as a comparison, we performed FISH experiments using well-established PNA probes. Then, DNA-PAINT imaging of telomeres and centromeres in the nucleus was performed using DNA strands as the probes. We also imaged the telomeres of AZT-treated HeLa cells to show the potential of DNA-PAINT in measuring telomere length.

## 2 Results and Discussion

### 2.1 Experimental Principle and Design

**Figure 1:**
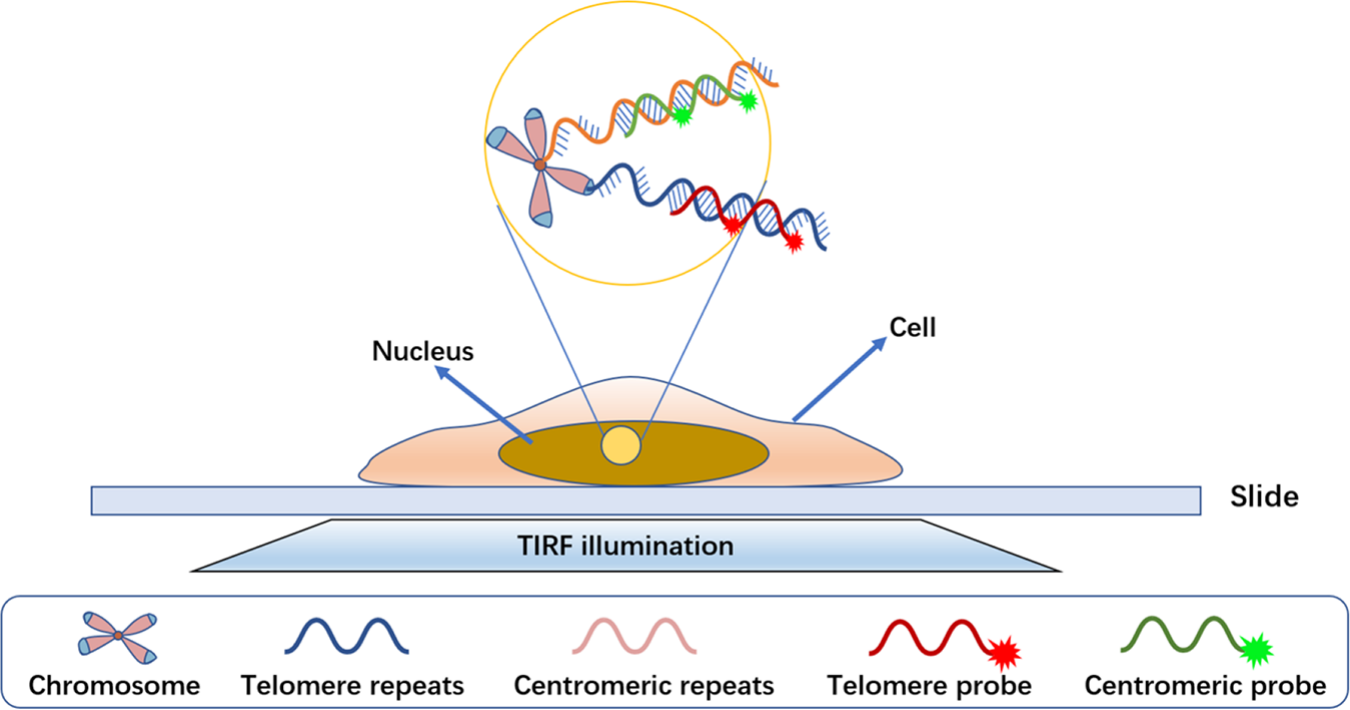
Schematic illustration of the working mechanism of DNA-PAINT-based in situ super-resolution imaging of telomeres and centromeres in cells.

Figure 1 shows the working mechanism of DNA-PAINT-based in situ imaging of centromeres and telomeres in the cell chromosomes. HeLa cells are first immobilized on the bottom of the chamber slide. The telomere DNA probe, which is used as the imager strand in DNA-PAINT, is modified with Cy5 and contains two CCCTAA repeats complementary to the telomeric repeats (TTAGGG). In order to ensure the active fluorescent blinking of the probe, the sequence of the telomere probe should not be too long. Therefore, the telomere probe contains two CCCTAA repeats. The centromere is another special structure on chromosomes that contains repetitive short DNA sequences, which could be used as an inner control since its length does not change like the telomere. Similarly, the centromere probe contains sequences complementary to centromere DNA and is modified with Cy3. Due to the limited illumination depth of the total internal reflection microscope (TIRF), only weak background noise is detected when the imager probes are suspended in buffer. If the imager probes enter the nucleus and bind to telomeres or centromeres, fluorescence of Cy5 or Cy3 could be detected, resulting in a fluorescence “on” signal. If the imager probes dissociate with telomeres or centromeres, a fluorescence “off” signal would be detected. Owing to the dynamic hybridization and dissociation of the short imager probes, photoswitching is realized, allowing DNA-PAINT imaging.

**Figure 2:**
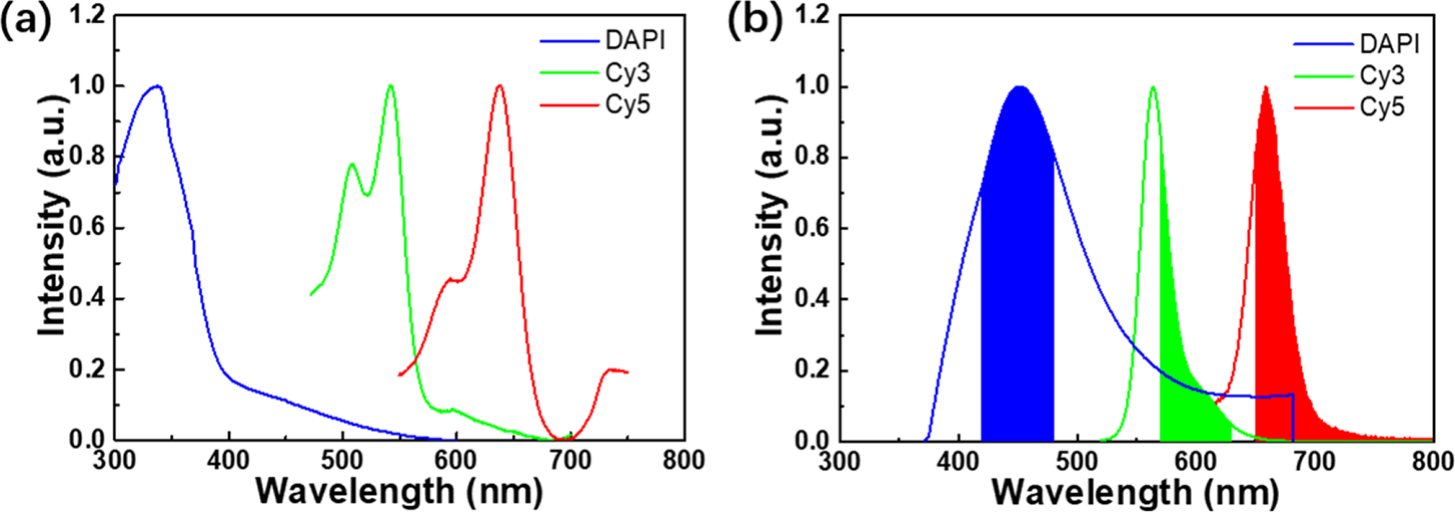
(a) Extinction spectra of DAPI, the telomere probes (Cy5), and the centromere probes (Cy3). (b) Fluorescence spectra of DAPI, the telomere probes (Cy5), and the centromere probes (Cy3).

In addition to Cy3- and Cy5-labeled imager probes, we also utilized DAPI to stain the cells. DAPI is a mature nuclear fluorescent dye that can penetrate the intact cell membrane and bind strongly to DNA in the cell nucleus. We stained cells with DAPI to facilitate the location of the nucleus. Figure 2a,b shows the extinction and fluorescence spectra of DAPI and the two DNA probes. Considering the lasers equipped with the microscope, we used 405, 561, and 642 nm lasers to excite DAPI, the centromere probes, and the telomere probes, respectively. The emission peaks of DAPI, the centromere probes, and the telomere probes are 451, 564, and 659 nm, respectively. According to the fluorescence spectra, the filter sets for DAPI, the centromere probes and the telomere probes are bandpass 420–480 nm, bandpass 570–630 nm, and longpass 655 nm, respectively. There is very little overlapping of their fluorescence spectra in these regions, resulting in negligible cross-talk of the signals from the three channels.

### 2.2 Intracellular Super-Resolution Imaging of the Telomere

Before DNA-PAINT-based super-resolution imaging of telomeres and centromeres, we performed FISH experiments using well-established PNA probes for comparison. In the past two decades, FISH technology has made great progress and plays an important role in the field of molecular cytogenetics.^26^ FISH can be used to study the localization of chromosomal DNA sequences, chromatin fiber FISH, DNA microarray quantification, and nuclear RNA expression analysis. FISH probes can bind directly to fluorescein-labeled nucleotides, simplifying label detection steps. At present, many kinds of FISH probes have been developed, such as gene probes, oligonucleotide probes, cDNA probes, and PNA probes. Among these, PNA probes are the most widely used ones due to excellent specificity.

**Figure 3:**
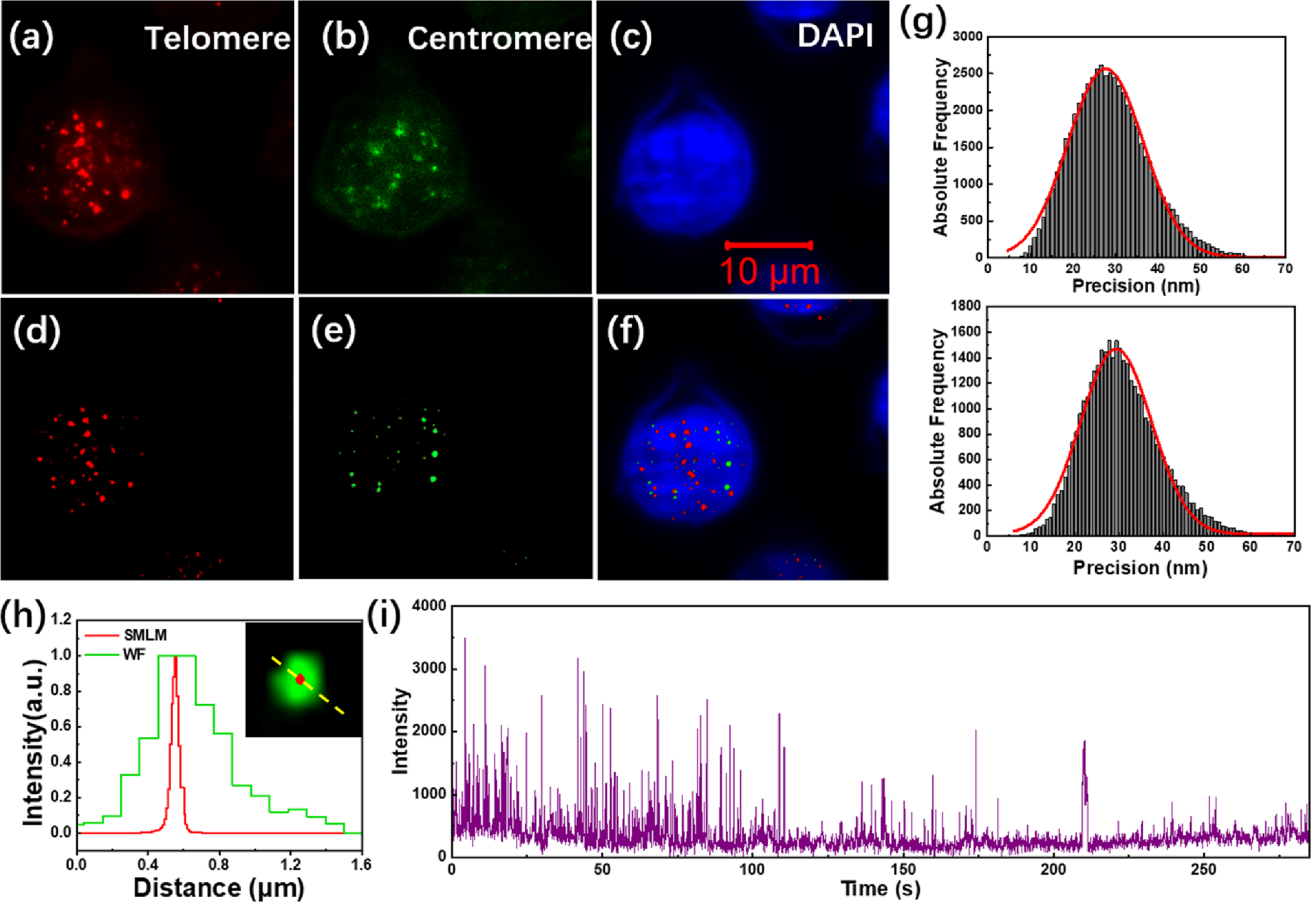
Imaging of telomeres and centromeres in HeLa cells using PNA probes. (a–c) Widefield TIRF images of the telomeres, centromeres, and nucleus of a HeLa cell. (d) SMLM image of telomeres of the same cell. (e) SMLM image of centromeres of the same cell. (f) Merged image of (c) to (e). (g) Localization precisions of the telomere probes (up) and centromere probes (down). (h) Relative intensity profiles along the dashed yellow line obtained from the WF image and SMLM image of the same telomere. (i) Time-dependent intensity profile of a single telomere where the telomere PNA probes bind to the telomere repeats.

FISH experiments were conducted according to the manufacturer’s manual. The telomere PNA probes were modified with Cy5, and the centromere PNA probes were also modified with Cy3. Therefore, the filter sets for the PNA probes were the same to those for the DNA probes. Wide-field TIRF images of telomere probes, centromere probes, and DAPI channels are shown in Figure 3a–c. The fluorescence signals of the telomere and centromere probes are basically distributed in the region of the nucleus, and there is no probe signal collected outside the nucleus, indicating excellent specificity of the PNA probes. Then, we performed SMLM imaging on the same sample. Compared with the wide-field TIRF image, the reconstructed SMLM image has higher signal-to-noise ratio and spatial resolution (Figure 3d,e). Figure 3f also shows good specificity of the PNA probes. The localization precisions of the telomere and centromere PNA probes are shown in Figure 3g, which are about 28 and 30 nm, respectively. According to Figure 3h, the full width at half maximum (FWHM) of the telomere obtained from the widefield (WF) and SMLM images are 500 and 34 nm, respectively. Compared to the widefield images, SMLM images have higher resolution.

However, during the imaging process, we discovered that the overall fluorescence intensity of the widefield image decreased continuously even with a special imaging buffer prepared according to previously published literature.^27^ For the time-dependent fluorescence intensity distribution curve of a single telomere obtained using PNA probes (Figure 3i), it can be seen that the blinking frequency of the telomere probe was very fast at the beginning but slowed down later. In addition, the observed fluorescence intensity was also decreasing. In the FISH experiment based on PNA probes, the redundant PNA probes are washed away, leaving only the probes that are hybridized to the telomeres and centromeres. During the SMLM imaging, the high-power excitation light and special imaging buffers are adopted so that the hybridized probes show photoswitching behavior. Due to the limited lifetime of fluorescent dyes, the dyes on the imaging probes are gradually consumed as the laser irradiation time increases. Therefore, unlike DNA-PAINT, there is no extra PNA probes to supplement during the SMLM imaging based on PNA probes, and the fluorescence intensity and flickering frequency gradually decrease, making it impossible to perform long-term imaging. Considering the expensive cost of PNA probes, we propose to use inexpensive DNA imaging probes to replace PNA probes and use DNA-PAINT to replace SMLM. Besides the cheap probes, DNA-PAINT imaging does not require high-power lasers to make the dye blink, so the lifetime of the dye and the imaging time are extended. In addition to the high excitation light power, DNA-PAINT imaging does not need a 405 nm activation light, so the excitation process of DNA-PAINT is much simple than SMLM.

### 2.3 In Situ Super-Resolution Imaging of Telomeres and Centromeres Using DNA-PAINT

**Figure 4:**
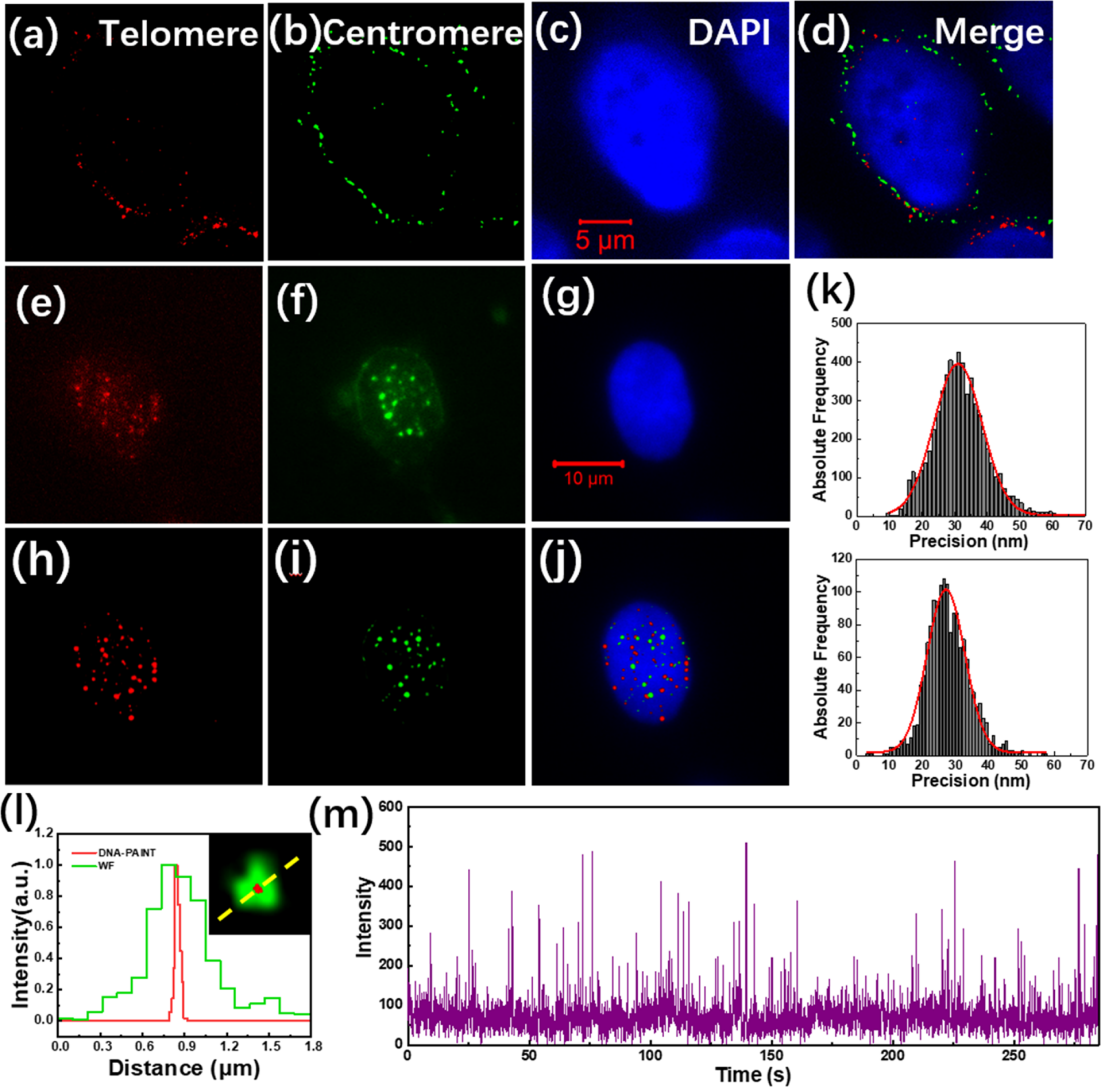
Imaging of telomeres and centromeres in HeLa cells using DNA probes. (a–d) HeLa cells permeabilized only once according to the FISH protocol. DNA-PAINT images of telomere probes (a) and centromeric probes (b), TIRF image of DAPI stained nucleus (c), merged image of (a) to (c) (d). (e–j) HeLa cells permeabilized twice according to the improved protocol. TIRF images of the telomeres (e), centromeres (f), and nucleus (g); DNA-PAINT images of the telomeres (h), centromeres (i), and merged image of (g) to (i) (j). (k) Localization precision of the telomere probes (up) and centromere probes (down). (l) Relative intensity profiles along the dashed yellow line obtained from WF and SMLM images of the same telomere. (m) Time-dependent intensity profile of a single site where the telomere DNA probes bind to the telomere repeats.

After testing the imaging effect of PNA probes, we performed DNA-PAINT-based imaging of the telomeres and centromeres. HeLa cells were first pretreated according to the procedures utilized for FISH, including the steps of fixation, permeabilization, RNA inhibition, BSA blocking, nuclear staining, pepsin digestion, and gradient dehydration (detailed information are shown in the Experimental Section). After pretreatment, hybridization buffer containing telomere probes was added to the chamber for the DNA-PAINT imaging process. To simultaneously obtain a good specificity and reversible binding between the telomere probe and the telomeres, the telomere probe (5′-Cy5-TTTTTCCCTAACCCTAA-3′) contains two CCCTAA repeats. TIRF images of the DAPI-stained nucleus and DNA-PAINT images of the telomere and centromere DNA probe channels are shown in Figure 4a–d. It can be seen that most of the fluorescence signals were observed on the edge of the nucleus and there is little fluorescence signal in the nucleus. This indicates that our probe rarely entered the nucleus and the permeabilization of the nuclear membrane was not good enough. Compared with the PNA probes, it is more difficult for the DNA probes to enter the nucleus; the main reason is the electrostatic repulsion between the DNA probes and the nucleus.

However, for DNA-PAINT imaging, it is pivotal for the DNA probes to penetrate the cell membrane and the nuclear membrane to enter the nucleus. Therefore, for a better penetration effect of the DNA probes, we used an new method we proposed before, which can better permeabilize the membranes.^28^ First, a lower concentration of Triton solution (0.2% w/w) was added into the cell chambers to perform a fast permeabilization of the membrane for only 45 s. Then, after the chambers were washed twice with PBS solution, the cells were fixed with paraformaldehyde. Next, a higher concentration of Triton solution (1% w/w) was added for a longer permeation time of 30 min. Finally, imager probes were added for DNA-PAINT imaging. Using this approach, after two rounds of permeabilization, the imager probes became more accessible to the cellular genome. Figure 4e–j shows the advantage of the improved experimental protocol. Many fluorescent sites can be observed in the nucleus, and there is basically no strong fluorescence signal outside the nucleus (Figure 4e–g). It shows that owing to the improved permeabilization strategy, the DNA probes can penetrate into the nucleus and specifically bind to their targets. During DNA-PAINT imaging, free probes in the imaging buffer randomly bind and dissociate from the target DNA sequences, resulting in the switching of fluorescence signals between ″on″ and ″off″ states. In the DNA-PAINT images of the telomeres (Figure 4h) and centromeres (Figure 4i), individual telomeres and centromeres can be clearly distinguished, showing much higher spatial resolution than the wide-field images. Figure 4j shows the merged images of the nucleus, telomeres, and centromeres, demonstrating the good specificity of the telomere and centromere probes. The localization precisions of the DNA probes are shown in Figure 4k, which are about 30 and 28 nm, respectively. The localization accuracy of the DNA probes was comparable to that of the PNA probes (Figure 3g). According to Figure 4l, the full width at half maximum (FWHM) of the telomere obtained from the widefield (WF) and DNA-PAINT images are about 420 and 37 nm, respectively. Compared to the widefield images, the DNA-PAINT images have higher resolution.

The light-switching behavior of the DNA imager probes was also investigated. For the time-dependent fluorescence intensity curve of telomere DNA probes at a single telomere binding site (Figure 4m), we could observe an obvious transient variation of the fluorescence intensity between the “on” and ″off″ states. Compared with the photoswitching of PNA probes shown in Figure 3i, we were always able to observe frequent blinking events, and the photoswitching frequency did not decrease significantly during the imaging process. The reason is that free imager strands can continuously bind and dossicate with the telomeres or centromeres. Moreover, we only needed to excite the fluorescence of the probes with a low laser power, so the fluorescence quenching phenomenon was not obvious. Therefore, the DNA-PAINT-based imaging strategy can image telomeres and centromeres for extended periods of time.

### 2.4 DNA-PAINT Imaging of Telomeres in Telomerase Inhibitor Treated Cells

To further verify the utility of our method, we added a telomerase inhibition drug (AZT) to HeLa cells and utilized DNA-PAINT imaging to evaluate its effect on telomere length. Telomerase, a special DNA polymerase that adds telomeric repeats to the ends of telomeric DNA, exhibits significant functional expression in most spontaneously immortalized cells. HeLa cells used in our experiments are tumor cells that highly express telomerase. The activity of telomerase enables HeLa cells to maintain sufficient telomere length during proliferation. AZT is a drug that inhibits the activity of telomerase, making the telomere length gradually decrease during proliferation.^29^ Theoretically, due to the inhibition of telomere length by AZT, the number of binding sites of telomere DNA probes is also reduced, resulting in a relatively inactive probe fluorescence compared to untreated HeLa cells.

**Figure 5:**
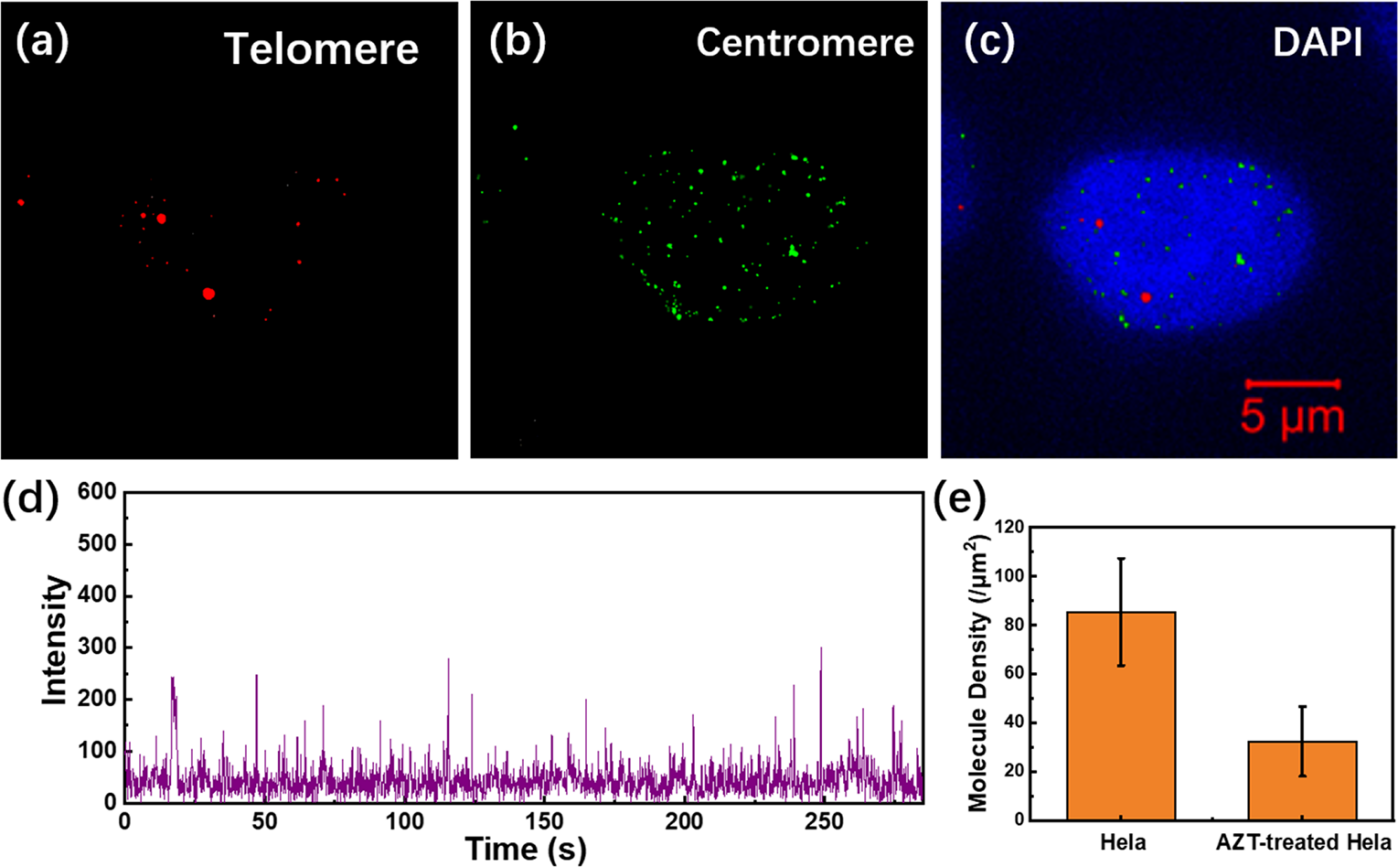
DNA-PAINT imaging of AZT treated HeLa cells. Telomeres (a), centromeres (b), and merged image of (a), (b), and the nucleus (c). (d) Time-dependent intensity profile of a single telomere site. (e) The mean molecular densities of telomeres obtained from the DNA-PAINT images of HeLa cells and AZT-treated Hela cells. The error bar is the standard deviation calculated from the molecular densities of seven samples.

In the experiment, we first treated HeLa cells with AZT. Then, DNA-PAINT imaging of the telomeres and centromeres of these cells was conducted. The DNA-PAINT images were provided in Figure 5. As can be seen, for telomeres, the fluorescence of the telomere probes was quite weak. There are significantly fewer telomeres in AZT-treated cells (Figure 5a) as compared with the untreated cells (Figure 4h). On the contrary, the signals of the centromeres (Figure 5b) did not change much as compared with untreated cells (Figure 4i). This is reasonable since AZT does not influence the centromere length.

By analyzing the time-dependent fluorescence intensity distribution curve (Figure 5d), the blinking activity of the AZT-treated HeLa cells was significantly attenuated as compared with the untreated ones (Figure 4m). Moreover, the fluorescence intensity of the imaging site was also obviously reduced compared to the untreated sample (Figure 4m). These observations were in line with our expectations. To better obtain quantitative results, the mean molecular density of the telomeres in the DNA-PAINT images was calculated using Zeiss ZEN software. The average molecular density obtained from the telomeres in untreated HeLa cells was found to be approximately 3 times higher than that in AZT-treated cells (Figure 5e). The inhibition of telomerase by AZT shortens the length of the telomeres, so the probability of hybridization between telomere probes and telomeres was also reduced, resulting in reduced molecular density.

In future work, we will perform DNA-PAINT experiments on synthetic telomeres of known lengths to obtain time-dependent scintillation curves. The calibration relationship between telomere length and blinking frequency or dark state time can be obtained by qPAINT analysis. Applying this calibration curve to our experiment, we can measure the telomere length in the nucleus and evaluate the inhibitory effect of AZT on telomere length more accurately. In this experiment, no treatment step is specifically designed for HeLa cells during cell treatment. The methods of cell fixation, penetration, and staining are also applicable to other cell lines. Hence, such a strategy has good general applicability toward other cell lines.

## 3 Conclusions

In this work, we demonstrated the first application of DNA-PAINT-based in situ super-resolution imaging of intracellular telomeres and centromeres. To facilitate the entrance of DNA imager probes into the cell nucleus, we utilized an improved permeabilization strategy. The spatial resolution of DNA-PAINT-based imaging of telomeres and centromeres is about 30 nm, well beyond the diffraction limit. We also demonstrated that DNA-PAINT could vividly reveal the telomerase inhibition effect of AZT. Although SMLM imaging of telomeres and centromeres could also be realized with PNA probe-based FISH, the DNA-PAINT strategy has several advantages. First, DNA imager probes are much cheaper than PNA probes. Second, SMLM imaging with PNA probes needs a special imaging buffer containing toxic reagents (e.g. mercaptoethanol), while DNA-PAINT imaging does not. Third, SMLM imaging requires a high excitation laser power and an activation laser (405 nm), while DNA-PAINT only needs one excitation laser with low power. More importantly, with the combination of qPAINT, the DNA-PAINT-based strategy would be able to directly measure the absolute length (i.e., base numbers) of telomere repeats with high precision, which we are planning to study in future work. We anticipate that such an advantageous DNA-PAINT imaging strategy has a great potential in the investigation of telomerase-related diseases.

## 4 Experimental Section

### 4.1 Materials

Phosphate-buffered saline (PBS, 10 mM, pH 7.4), Tween 20, RNA inhibitor (RNAse A), pepsin, Triton X-100, formamide, 4% paraformaldehyde, salmon sperm DNA, and DAPI were purchased from Nanjing KeyGen Biotechnology Co., Ltd. Tris and ethanol (100%) were purchased from Sinopharm Chemical Reagent Co., Ltd. Sodium chloride (NaCl) and hydrochloric acid (HCl) were purchased from Guangdong Xilong Chemical Co., Ltd. Glucose oxidase, catalase, and beta-mercaptoethanol (βME) were purchased from Sigma-Aldrich. The reagents used in the experiment do not need further purification operations, and the deionized water used in the experiment is 18.25 MΩ/cm (Millipore Milli-Q grade) pure water. Peptide nucleic acid (PNA) probes for FISH were purchased from Panagene (Korea), including Cy5 dye-labeled telomere PNA probe F1003 and Cy3 dye-labeled centromere PNA probe F3003. Oligonucleotides were synthesized by Sangon Biotech (Shanghai) Co., Ltd. The sequences are as follows:
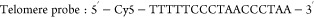

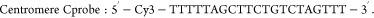


### 4.2 Cell Culture and AZT Inhibition

Human cervical cancer cells (HeLa) were purchased from the Cell Bank of Shanghai Institutes for Biological Sciences, Chinese Academy of Sciences. The HeLa cell culture medium is a mixture of 90% DMEM high glucose, 10% fetal bovine serum (GIBCO), and 1% penicillin–streptomycin. DMEM high glucose and penicillin mixture were purchased from Nanjing KGI Biotechnology Co., Ltd. The cell culture conditions are 5% carbon dioxide atmosphere, and the culture temperature is constant at 37 °C.

In our experiments, cells treated with AZT were cultured for four to six passages. In order to significantly verify the inhibitory effect of the inhibitor on telomere length, the treated cells need to be cultured for at least 16 passages before experimental verification in further studies.

### 4.3 Cell Pretreatment of FISH Experiment Based on PNA Probes

First, 4% paraformaldehyde fixative solution was added to the cell culture plate inoculated with the cells to be tested for 30 min and rinsed with PBS solution to remove excess paraformaldehyde. Then, 1% (w/w) Triton X-100 solution was added to penetrate the cell membrane for 30 min. RNA inhibitor A (RNase A, 100 μg/mL) was added for RNA digestion for 45 min. After that, DAPI was added for 20 min to stain the nucleus. Then, 0.005% (w/w) pepsin solution was added to decompose the protein for more than 5 min in 37 °C. The pepsin solution was prepared using dilute hydrochloric acid with a concentration of 10 mM. Before adding the pepsin solution, it was preheated to 45 °C, and the dilute hydrochloric acid was also heated to 37 °C before preparation. Ice ethanol with concentrations of 70, 85, and 100% (w/w) were added to dehydrate the cells in a gradient, and each dehydration time was 2 min.

### 4.4 Cell Pretreatment of DNA-PAINT Experiment Based on DNA Probes

First, 0.2% (w/w) Triton X-100 solution was added to penetrate the cell membrane for 45–60 s. Then, the slide was washed with PBS solution gently 3 times. Next, 4% paraformaldehyde fixative solution was added to the cell culture plate inoculated with the cells for 30 min. The cells were rinsed with PBS solution to remove excess paraformaldehyde. Then, 1% (w/w) Triton X-100 solution was added to penetrate the cell membrane for 30 min. RNA inhibitor A (RNase A, 100 μg/mL) was added for RNA digestion for 45 min. After that, DAPI was added for 20 min to stain the nucleus. Then, 0.005% (w/w) pepsin solution was added to decompose the protein for more than 5 min in 37 °C. The pepsin solution is prepared using dilute hydrochloric acid with a concentration of 10 mM. Before adding the pepsin solution, it was preheated to 45 °C, and the dilute hydrochloric acid was also heated to 37 °C before preparation. Ice ethanol with concentrations of 70, 85, and 100% (w/w) were added to dehydrate the cells in a gradient, and each dehydration time was 2 min.

### 4.5 Fluorescence Imaging of Telomeres and Centromeres

Before adding the imaging probes, the slide was incubated in 85 °C for 5 min.

For the FISH experiment, the PNA probes were dissolved into hybridization buffer (20 mM Tris–HCl (pH 7.4), 60% formamide, 0.1 μg/mL salmon sperm DNA, and 100 mM NaCl) with a final concentration of 1 nM. Then, the solution was added, and the slide was incubated in 85 °C for 10 min and slowly cooled down to room temperature. After that, the slide was incubated for 3 h in 37 °C. Next, the slide was washed with a wash solution (2× SSC, 0.1% Tween 20) twice at 55–60 °C for 10 min. Finally, imaging buffer (5 mg/mL glucose oxidase, 0.5 mg/mL catalase, 10% βME, and 10 mM PBS) was added to the chamber.

For the DNA-PAINT experiment, the telomere probes and centromere probes were dissolved into hybridization buffer with a final concentration of 1 nM. Then, the solution was added, and the slide was incubated in 85 °C for 10 min and slowly cooled down to room temperature. Finally, the slide was incubated for 3 h in 37 °C. The entire reaction process was kept away from light.

### 4.6 Instruments

SMLM and DNA-PAINT images were acquired using a Zeiss Elyra P.1 microscope equipped with 405 nm (100 mW), 561 nm (100 mW), and 642 nm (100 mW) lasers, a 100×/1.46 oil immersion objective, and an Andor EM-CCD camera (iXon DU897). SMLM images of the telomere probes were obtained using 405 and 642 nm excitation with a 655 nm longpass filter. SMLM images of the centromere probes were obtained using 405 and 561 nm excitation with a 570–630 nm bandpass filter. DNA-PAINT images of the telomere probes were obtained using 642 nm excitation with a 655 nm longpass filter. DNA-PAINT images of the centromere probes were obtained using 561 nm excitation with a 570–630 nm bandpass filter. The exposure time for SMLM was 50 ms, and that for DNA-PAINT was 150 ms.

Widefield TIRFM images of the DAPI dye was obtained using 405 nm excitation with a 420–480 nm bandpass filter; the exposure time for each frame was 150 ms. For each super-resolved SMLM or DNA-PAINT images, 5000 or 10,000 frames were collected for reconstruction. The super-resolved images were reconstructed using Zeiss ZEN 2012 software integrated with the microscope using the Gaussian fitting of each blinking event. The parameters were kept constant for different samples.
